# Episode Charges and Subsequent Visits After Telemedicine vs In-Person Care

**DOI:** 10.1001/jamanetworkopen.2025.56127

**Published:** 2026-02-09

**Authors:** Bingyu Zhang, Lu Li, Yiwen Lu, John B. Salmon, Robert L. Stetson, Michael A. Horst, Xuan Bi, Srinivas K. Sridhara, Mitchell D. Schnall, Tessa S. Cook, Irene Papanicolas, Shivan J. Mehta, Kevin B. Mahoney, David A. Asch, Yong Chen

**Affiliations:** 1 Center for Health AI and Synthesis of Evidence (CHASE), University of Pennsylvania, Philadelphia; 2 The Graduate Group in Applied Mathematics and Computational Science, School of Arts and Sciences, University of Pennsylvania, Philadelphia; 3 University of Pennsylvania Health System, Philadelphia; 4 Carlson School of Management, University of Minnesota, Minneapolis; 5 Department of Radiology, Perelman School of Medicine, University of Pennsylvania, Philadelphia; 6 Department of Health Services, Policy, and Practice, Brown University School of Public Health, Providence, Rhode Island; 7 Department of Medicine, Perelman School of Medicine, University of Pennsylvania, Philadelphia; 8 Penn Center for Health Incentives and Behavioral Economics, University of Pennsylvania, Philadelphia; 9 Leonard Davis Institute for Health Economics, University of Pennsylvania, Philadelphia; 10 Center for Health Care Transformation and Innovation, University of Pennsylvania, Philadelphia; 11 Office of the Chief Executive Officer, University of Pennsylvania Health System, Philadelphia; 12 Wharton School, University of Pennsylvania, Philadelphia; 13 Division of General Internal Medicine, University of Pennsylvania, Philadelphia; 14 Department of Biostatistics, Epidemiology, and Informatics, University of Pennsylvania Perelman School of Medicine, Philadelphia; 15 Penn Medicine Center for Evidence-Based Practice (CEP), Philadelphia, Pennsylvania; 16 Penn Institute for Biomedical Informatics (IBI), Philadelphia, Pennsylvania

## Abstract

**Importance:**

Telemedicine use increased during the COVID-19 pandemic and has remained a regular component of health care delivery. However, the financial implications of this change for health systems’ reimbursement and utilization remain unclear.

**Objective:**

To compare 30-day episode charges and subsequent visits after telemedicine and in-person index visits.

**Design, Setting, and Participants:**

The target trial emulation conducted in this comparative effectiveness research included ambulatory in-person and telemedicine visit data from an academic health system comprising 5 hospitals in Pennsylvania from January 1 to April 30, 2024. Analyses focused on 10 high-volume clinical conditions commonly managed through telemedicine.

**Exposures:**

Telemedicine visits vs in-person visits.

**Main Outcomes and Measures:**

Outcomes included episode charges (the billed amount submitted for reimbursement to insurers and patients, excluding physician professional and facility fees for the index encounter) in an episode window from 7 days before to 30 days after the index visit and the number of subsequent visits within the episode window. Linear regression and Poisson regression with propensity score matching were conducted to adjust for demographic, clinical, socioeconomic, and contextual factors.

**Results:**

A total of 163 308 visits (108 383 [66.4%] among females; mean [SD] patient age, 49.2 [19.1] years) were included in this study. After propensity score matching, the mean 30-day episode charge was $96.60 (95% CI, $92.24-$100.96) for telemedicine encounters and $509.21 (95% CI, $500.65-$517.77) for in-person encounters (mean difference, $412.62; 95% CI, $403.01-$422.22). Additionally, telemedicine visits were associated with fewer follow-up visits per 30-day episode than were in-person visits (mean [SD], 3.44 [5.38] vs 4.44 [7.41] visits; comparative reduction, 23% [95% CI, 20%-26%]). For mental and behavioral disorders, 3 categories—depressive disorders (−$69.47; 95% CI, −$100.90 to −$38.04), anxiety and fear-related disorders ($38.06; 95% CI, $23.14 to $52.99), and neurodevelopmental disorders (−$28.88; 95% CI, −$54.72 to −$3.04)—exhibited comparable episode charges for telemedicine vs in-person encounters.

**Conclusions and Relevance:**

In this comparative effectiveness research using target trial emulation of outpatient telemedicine and in-person visits, telemedicine visits overall were associated with lower charges and fewer subsequent visits within the 30-day episode than were in-person visits. For mental and behavioral conditions, charges were comparable. These findings suggest that telemedicine may serve as a lower-charge alternative to in-person care without increasing the need for subsequent visits.

## Key Points

**Question:**

Do charges and subsequent visits differ between telemedicine and in-person visits in the postpandemic period?

**Findings:**

In this comparative effectiveness research of a target trial emulation using data from 163 308 health care encounters, telemedicine visits overall were associated with lower charges and fewer subsequent visits within a 30-day episode compared with in-person visits. Episode charges for mental and behavioral disorders were comparable or even higher for telemedicine vs in-person visits.

**Meaning:**

These findings suggest telemedicine may cost less than in-person care without increasing short-term subsequent use and may enable more strategic resource allocation, reduce unnecessary use, and inform future reimbursement and policy decisions.

## Teaser

This comparative effectiveness research compares charges for and subsequent visits after telemedicine vs in-person health care encounters for 10 common clinical conditions within an academic health system.

## Introduction

Telemedicine use increased greatly during the COVID-19 pandemic, and while use has declined since, it remains higher than in prepandemic periods.^1,2^ At Penn Medicine, a large academic health system serving approximately 5 million patients across southeastern Pennsylvania and surrounding regions, telemedicine adoption was swift.^3^ Between 2020 and 2022, over 2.1 million virtual visits were conducted. During the 2024 study period, Penn Medicine recorded over 3 million outpatient encounters, of which approximately 4% were delivered via telemedicine, reflecting the system’s sustained integration of virtual care into routine outpatient practice.^2,4^

Despite the persistent postpandemic use of telemedicine, its impact on overall health care charges, defined as the total amount submitted for payers and consumers and reimbursement by hospitals and other clinical settings, remains unclear. Prior work has primarily focused on comparing clinical outcomes or utilization patterns following telemedicine vs in-person visits, such as differences in follow-up rates, emergency department use, or hospitalizations.^5,6,7,8,9,10^ Only a few studies have examined cost-related outcomes using clinical health system data. For example, Chaiyachati et al^11^ and Ashwood et al^12^ analyzed employer-sponsored, direct-to-consumer telemedicine services and found that telemedicine may lower per-episode costs but marginally increase use. Faleh AlMutairi et al^13^ evaluated telemedicine for patients with uncontrolled type 2 diabetes and included clinical costs such as laboratory procedures, medications, and supplies. Despite slightly higher costs, telemedicine improved outcomes and was cost-effective overall. Saharkhiz et al^14^ found that higher levels of telehealth use may be associated with higher total cost of care among Medicare beneficiaries. These studies focused on fragmented payer types, but each was limited to a single insurance segment or specific patient population and therefore did not capture broader, systemwide financial implications across diverse outpatient encounters.

To address these gaps, we conducted a target trial emulation using electronic health records and billing data from Penn Medicine to assess how telemedicine compares with in-person care in outpatient settings across 10 common diagnostic categories in telemedicine. We evaluated 30-day financial (care episode charges) and utilization (number of subsequent visits) outcomes following the index encounter. By capturing reimbursed services over a defined 30-day episode of care, our study provides a health system–level perspective on how telemedicine affects cost and use and supports the development of sustainable models for virtual care delivery in the postpandemic era.

## Methods

### Data Source and Study Population

In this comparative effectiveness research, a target trial emulation was conducted using electronic health record (EHR) and billing data from 5 hospitals within Penn Medicine, including the Hospital of the University of Pennsylvania (HUP), Penn Presbyterian Medical Center (PPMC), Pennsylvania Hospital (PAH), Chester County Hospital (CCH), and Penn Medicine Princeton Medical Center (PMC). These hospitals serve a sociodemographically diverse population across southeastern Pennsylvania and neighboring regions, with approximately 5 million patients receiving care over the past decade. The study was reviewed and approved by the University of Pennsylvania institutional review board, which granted a waiver of informed consent because the study involved minimal risk and used deidentified retrospective EHR data. This study followed the Transparent Reporting of Observational Studies Emulating a Target Trial (TARGET) reporting guideline.^15^

The study period spanned January 1 to April 30, 2024. We included outpatient encounters conducted via telemedicine or in-person visits and excluded encounters originating in the emergency department or inpatient settings. We defined an index encounter as a telemedicine or in-person outpatient visit that initiated an episode of ambulatory care, representing a clinically meaningful face-to-face interaction with a clinician delivered either in person or via synchronous video. Encounters primarily designated for laboratory tests, radiologic imaging, medication refills, or telephone-only communication were excluded, as these are not typically used to initiate or manage an episode of care. Laboratory tests, imaging, medication refills, and procedures occurring before or after the index visit were included separately as downstream services within the episode window. A complete list of included visit types is provided in eTable 1 in Supplement 1.

Among the identified index encounters, we further restricted our sample to visits related to the 10 most common clinical concerns managed through telemedicine during the study period. These 10 diagnostic categories accounted for approximately 30% of all telemedicine visits during the study period, making them highly representative of clinical virtual care delivery. The selected categories span a diverse range of clinical domains, including mental and behavioral health, infectious diseases, endocrine and reproductive conditions, and symptom-based presentations, capturing high-volume, clinically relevant use cases across multiple body systems. Clinical concern categories were defined using the Clinical Classifications Software Refined (CCSR) from the Healthcare Cost and Utilization Project,^16^ which groups the *International Statistical Classification of Diseases and Related Health Problems, Tenth Revision (ICD-10)* diagnoses into clinically meaningful categories. To maintain comparability, in-person visits were restricted to the same set of CCSR primary diagnosis categories as telemedicine visits. The clinical areas included obesity, contraceptive and procreative management, COVID-19, depressive disorders, anxiety and fear-related disorders, neurodevelopmental disorders, sleep-wake disorders, other specified inflammatory conditions of skin, respiratory signs and symptoms, and abnormal findings without diagnosis. A detailed list of definitions is provided in eTable 2 in Supplement 1. The cohort construction process is illustrated in eFigure 1 in Supplement 1.

### Study Design

We conducted a target trial emulation to compare outcomes between telemedicine and in-person outpatient visits, mimicking the design of a hypothetical randomized trial. Each eligible encounter was analyzed using an intention-to-treat framework, with visit modality (telemedicine or in-person) defined at the index encounter (time 0). Patients could contribute multiple eligible encounters and appear in both the telemedicine and in-person groups at different times. Visits occurring within the window extending from 7 days before to 30 days after the visit, regardless of modality, were considered outcomes. The protocol specification and schematic timeline of the emulated trial are shown in eTable 4 and eFigure 2 in Supplement 1.

### Outcomes

The outcomes were episode charges and the number of subsequent health care visits within a 30-day episode window. The episode window was defined as 7 days prior to and 30 days following the index visit to capture a comprehensive view of care associated with the encounter. The 7-day previsit period was included to account for preparatory services, such as imaging and laboratory tests, that are often ordered in advance but billed separately from the visit itself. These thresholds were selected based on clinical input from practicing physicians and reflect typical care patterns observed in ambulatory settings. Including this period ensured that clinically related resource use was not underestimated.

Episode charges were measured as the billed amount: the total reimbursement submitted to the insurer and patient cost-sharing (such as copayments, coinsurance, or deductibles). We excluded physician professional fees and facility fees associated with the index encounter to ensure comparability of downstream costs across visit types, as these are often subject to different reimbursement schedules for telemedicine vs in-person visits and could introduce bias due to structural payment differences unrelated to care intensity or follow-up needs. These fees were identified and removed using Current Procedural Terminology and Healthcare Common Procedure Coding System codes, which are standardized coding systems used in the US for billing and processing health insurance claims.

Both charges and subsequent visits included nonprofessional services delivered within the episode window, such as laboratory tests, imaging, medication refills, procedures, and additional face-to-face encounters. These outcomes were used to assess differences in downstream resource use and care patterns between visit modalities.

### Patient Characteristics and Covariates

We assessed an extensive set of previsit characteristics as measured confounders to adjust for the potential confounding effect. Demographic variables included patient age at index date (<40, 40-64, or ≥65 years), sex (female, male), EHR-derived race and ethnicity (included in the analysis for confounding control given potential associations with health care access and utilization; categories were Asian, Hispanic, non-Hispanic Black [hereafter, Black], non-Hispanic White [hereafter, White], and other [included American Indian or Alaska Native, Native Hawaiian or Other Pacific Islander, multiracial, and unknown or not reported]), and marital status (married, unmarried). Clinical variables included the Charlson Comorbidity Index (CCI) score^17^ (0, 1-2, or ≥3 on a scale from 0-30, with higher scores indicating greater burden of comorbid disease) and CCSR-categorized primary diagnosis. Socioeconomic and contextual factors included insurance plan (commercial, Medicare, Medicaid, and self-pay or unknown), patient portal use (yes, no), zip code–level median household income from census data^18^ ($50 000, $50 000 to $100 000, or ≥$100 000), and distance from the patient’s residence to the place of service (<8 km, 8-24 km, or ≥24 km). We also adjusted for encounter type (new patient visit, return patient visit, or unknown), month of index visit (from January to April 2024), and indicators from the 5 data-contributing sites. A detailed list of study variables is provided in eTable 3 in Supplement 1.

### Statistical Analysis

We summarized baseline characteristics by visit modality using descriptive statistics, and differences were evaluated with χ^2^ tests to understand differences in characteristics by visit modality. To mitigate the effects of confounding factors, we used propensity score matching to adjust for the measured confounders collected before the index date.^19,20^ We fitted a logistic regression model by regressing the response variable (telemedicine or in-person visit) on demographic, clinical, and contextual covariates. The estimated probabilities from the logistic regression model gave the propensity score for each visit, representing its likelihood of belonging to the telemedicine visit group given the observed covariates. Visits were matched using up to 1:5 nearest neighbor matching without replacement, with a caliper of 0.2 pooled SDs. Some telemedicine visits had fewer than 5 matched in-person visits due to the caliper restriction and lack of replacement. Covariate balance was assessed using standardized mean differences, with a difference of 0.1 or less considered an acceptable balance.^21^

Within the matched cohort, the mean difference in 30-day episode charges was estimated using a linear regression model, and the relative risk for subsequent visits was estimated using the Poisson regression model. Statistical significance for model coefficients was evaluated using 2-sided *t* tests from each regression model, with 2-sided *P* < .05 considered statistically significant. Corresponding 95% CIs were also reported. To account for within-patient correlation arising from repeated visits, we used sandwich (robust) SEs clustered at the patient level.

We conducted several sensitivity analyses to examine the robustness of the results. First, we repeated the analyses using propensity score weighting instead of matching. Second, we repeated the primary analysis, including both professional and facility fees in the total 30-day episode charges. Additionally, we conducted subgroup analyses by repeating the target trial emulation within stratified subsets of the cohort, based on the data-contributing hospital and clinical conditions as defined by CCSR categories. Within each CCSR category, we further repeated the emulation across hospitals to evaluate the consistency of outcomes. Each analysis was performed independently using the same analytic framework.

All analyses were performed using R, version 4.3.1 (R Project for Statistical Computing). Missing categorical data were coded as other or unknown, and continuous variables were imputed using multiple imputation by chained equations.

## Results

### Cohort Identification

Between January 1 and April 30, 2024, Penn Medicine recorded 163 308 outpatient face-to-face health care encounters across the 10 CCSR categories, with a mean (SD) patient age of 49.2 (19.1) years: 29 446 (18.0%) via telemedicine and 133 862 (82.0%) in person. Of these, 54 925 (33.6%) were among men and 108 383 (66.4%) among women. A total of 9014 encounters (5.5%) were among Asian patients; 34 122 (20.9%), Black; 8813 (5.4%), Hispanic; 97 848 (60.0%), White; and 13 521 (8.3%), other race and ethnicity. Nearly half of encounters (48.0%) were among married patients, and 88.8% were among patient portal users. Telemedicine visits were more likely among patients aged 64 years or younger, commercially insured patients, portal users, unmarried patients, return patients, patients with lower CCI scores, and patients with longer distances to care. A detailed comparison of baseline characteristics between telemedicine and in-person visits is summarized in Table 1.

**Table 1.** Baseline Characteristics of Telemedicine and In-Person Visits Among Outpatient Encounters From January to April 2024

Characteristic	Encounters, No. (%)			*P* value^a^
	Telemedicine visits (n = 29 446)	In-person visits (n = 133 862)	Overall (N = 163 308)	
Visit month				
January	8363 (28.4)	32 804 (24.5)	41 167 (25.2)	<.001
February	7134 (24.2)	32 220 (24.1)	39 354 (24.1)	
March	6689 (22.7)	33 484 (25.0)	40 173 (24.6)	
April	7260 (24.7)	35 354 (26.4)	42 614 (26.1)	
Hospital				
CCH	258 (0.9)	12 714 (9.5)	12 972 (7.9)	<.001
HUP	22 086 (75.0)	64 081 (47.9)	86 167 (52.8)	
PMC	2045 (6.9)	17 625 (13.2)	19 670 (12.0)	
PAH	2862 (9.7)	23 272 (17.4)	26 134 (16.0)	
PPMC	2195 (7.5)	16 170 (12.1)	18 365 (11.2)	
Age range, y				
<40	13 313 (45.2)	45 513 (34.0)	58 826 (36.0)	<.001
40-64	10 951 (37.2)	50 738 (37.9)	61 689 (37.8)	
≥65	5182 (17.6)	37 611 (28.1)	42 793 (26.2)	
Sex				
Female	19 717 (67.0)	88 666 (66.2)	108 383 (66.4)	.02
Male	9729 (33.0)	45 196 (33.8)	54 925 (33.6)	
Race and ethnicity				
Asian	1441 (4.9)	7573 (5.7)	9014 (5.5)	<.001
Hispanic	1534 (5.2)	7279 (5.4)	8813 (5.4)	
Non-Hispanic Black	5584 (19.0)	28 528 (21.3)	34 112 (20.9)	
Non-Hispanic White	18 262 (62.0)	79 586 (59.5)	97 848 (60.0)	
Other^b^	2625 (8.9)	10 896 (8.1)	13 521 (8.3)	
Insurance plan				
Commercial	15 374 (52.2)	60 035 (44.8)	75 409 (46.2)	<.001
Medicaid	3425 (11.6)	14 960 (11.2)	18 385 (11.3)	
Medicare	4464 (15.2)	28 906 (21.6)	33 370 (20.4)	
Self-pay or unknown	6183 (21.0)	29 961 (22.4)	36 144 (22.1)	
MyPennMedicine patient portal user				
No	591 (2.0)	17 750 (13.3)	18 341 (11.2)	<.001
Yes	28 855 (98.0)	116 112 (86.7)	144 967 (88.8)	
Marital status				
Married	12 995 (44.1)	65 312 (48.8)	78 307 (48.0)	<.001
Not married	16 451 (55.9)	68 550 (51.2)	85 001 (52.0)	
Encounter category				
Return patient visit	25 411 (86.3)	82 816 (61.9)	108 227 (66.3)	<.001
New patient visit	4031 (13.7)	18 519 (13.8)	22 550 (13.8)	
Unknown	4 (0.0)	32 527 (24.3)	32 531 (19.9)	
Median household income, $				
<50 000	6025 (20.5)	25 703 (19.2)	31 728 (19.4)	<.001
50 000-100 000	14 536 (49.4)	63 053 (47.1)	77 589 (47.5)	
≥100 000	8885 (30.2)	45 106 (33.7)	53 991 (33.1)	
CCI score^c^				
0	12 945 (44.0)	50 683 (37.9)	63 628 (39.0)	<.001
1-2	8487 (28.8)	34 122 (25.5)	42 609 (26.1)	
≥3	8014 (27.2)	49 057 (36.6)	57 071 (34.9)	
Distance from home to place of service, km				
<8	12 921 (43.9)	59 222 (44.2)	72 143 (44.2)	<.001
8-24	9588 (32.6)	51 938 (38.8)	61 526 (37.7)	
≥24	6937 (23.6)	22 702 (17.0)	29 639 (18.1)	

Abbreviations: CCH, Chester County Hospital; CCI, Charlson Comorbidity Index; HUP, Hospital of the University of Pennsylvania; PAH, Pennsylvania Hospital; PMC, Penn Medicine Princeton Medical Center; PPMC, Penn Presbyterian Medical Center.

^a^ Pearson χ^2^ test.

^b^ Includes American Indian or Alaska Native, Native Hawaiian or Other Pacific Islander, multiracial, and unknown or not reported.

^c^ Score range, 0 to 30, with higher scores indicating greater burden of comorbid disease.

### Distribution by Condition

Table 2 presents the distribution of index encounters across the 10 CCSR categories, stratified by modality. The proportion of telemedicine use varied by clinical condition. Categories such as COVID-19 (67.3%), anxiety and fear-related disorders (34.6%), depressive disorders (30.0%), and sleep-wake disorders (33.1%) had relatively high rates of telemedicine visits, reflecting their suitability for remote care delivery. In contrast, lower rates of telemedicine use were observed for respiratory signs and symptoms (10.3%), obesity (18.2%), neurodevelopmental disorders (23.0%), and abnormal findings without diagnosis (4.3%).

**Table 2.** Distribution of Telemedicine and In-Person Visits Across 10 Common CCSR Categories

CCSR code	Description (total No. of patients)	Patients, No. (%)	
		Telemedicine visits	In-person visits
END009	Obesity (n = 9189)	1671 (18.2)	7518 (81.8)
FAC013	Contraceptive and procreative management (n = 14 676)	1750 (11.9)	12 926 (88.1)
INF012	COVID-19 (n = 3244)	2182 (67.3)	1062 (32.7)
MBD002	Depressive disorders (n = 17 008)	5094 (30.0)	11 914 (70.0)
MBD005	Anxiety and fear-related disorders (n = 19 420)	6710 (34.6)	12 710 (65.4)
MBD014	Neurodevelopmental disorders (n = 5998)	1381 (23.0)	4617 (77.0)
NVS016	Sleep-wake disorders (n = 15 563)	5147 (33.1)	10 416 (66.9)
SKN002	Other specified inflammatory conditions of skin (n = 13 759)	1282 (9.3)	12 477 (90.7)
SYM013	Respiratory signs and symptoms (n = 24 324)	2513 (10.3)	21 811 (89.7)
SYM017	Abnormal findings without diagnosis (n = 40 127)	1716 (4.3)	38 411 (95.7)

Abbreviation: CCSR, Clinical Classifications Software Refined.

### Overall 30-Day Episode Charges and Subsequent Visits

**Figure 1.**
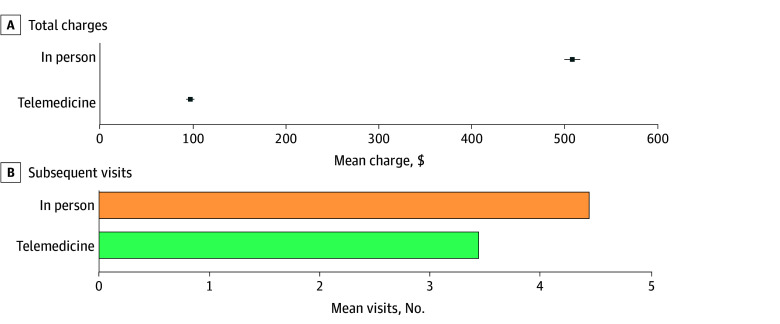
Graph Comparing 30-Day Episode Charges and Number of Subsequent Visits After Telemedicine and In-Person Index Visits Total charges represent the sum of billed amounts submitted for reimbursement to insurer and patient payments. Error bars denote 95% CIs.

After propensity score matching, 27 541 telemedicine visits were matched to 72 481 in-person visits with well-balanced characteristics (eFigure 3 in Supplement 1). Figure 1 displays comparisons of total charges and the number of subsequent visits per 30-day episode after matching. The mean total charge for telemedicine visits was $96.60 (95% CI, $92.24-$100.96), substantially lower than the $509.21 (95% CI, $500.65-$517.77) observed for in-person visits. The mean difference was $412.62 (95% CI, $403.01-$422.22; *P* < .001).

In addition, a mean (SD) of 3.44 (5.38) subsequent visits occurred after telemedicine encounters, compared with 4.44 (7.41) after in-person visits. This corresponds to a 23% (95% CI, 20%-26%) comparative reduction in 30-day subsequent visits (*P* < .001). Sensitivity analyses, using propensity score weighting (eTable 5 in Supplement 1) and including physician and facility fees (eTable 6 in Supplement 1), yielded consistent results with the primary findings.

### Site-Level Stratification

**Figure 2.**
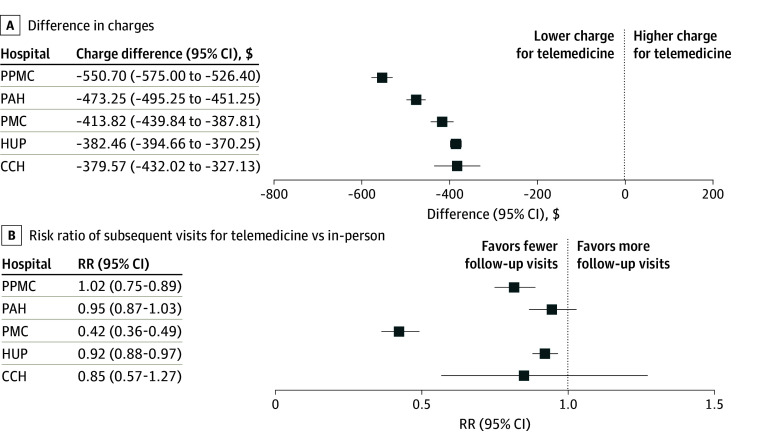
Forest Plots of Hospital-Specific Differences in 30-Day Episode Charges and Subsequent Visits After a Telemedicine vs In-Person Visit A, Charge differences were measured as telemedicine cost minus in-person cost in dollars. B, Risk ratios (RRs) were measured as the mean number of subsequent visits after telemedicine vs in-person visits. CCH indicates Chester County Hospital; CCSR, Clinical Classifications Software Refined; HUP, Hospital of the University of Pennsylvania; PAH, Pennsylvania Hospital; PMC, Penn Medicine Princeton Medical Center; PPMC, Penn Presbyterian Medical Center.

Figure 2 shows hospital-specific comparisons of 30-day episode charges and subsequent visit rates. Telemedicine was associated with lower charges across all 5 hospitals. The largest difference was observed at PPMC, where the incurred cost per episode of telemedicine visits minus in-person visits was −$550.70 (95% CI, −$575.00 to −$526.40), followed by PAH (−$473.25; 95% CI, −$495.25 to −$451.25). Even sites with comparatively smaller differences, such as CCH and HUP, showed consistent charge reductions.

Telemedicine was also associated with fewer subsequent visits across most hospitals. PMC had the largest reduction (risk ratio [RR], 0.42; 95% CI, 0.36-0.49). More modest reductions were seen at PAH (RR, 0.95; 95% CI, 0.87-1.03) and HUP (RR, 0.92; 95% CI, 0.88-0.97).

### Condition-Level Stratification

**Figure 3.**
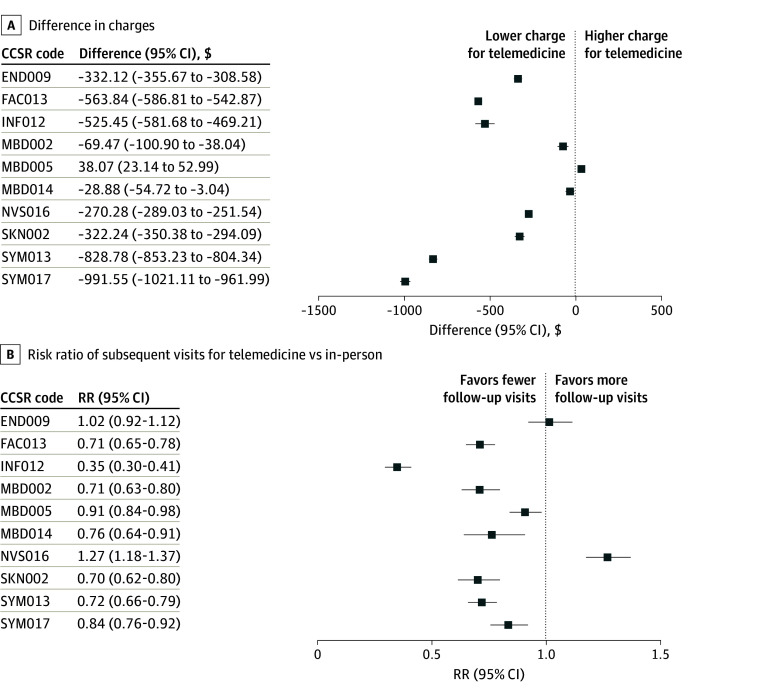
Forest Plots of Condition-Specific Differences in 30-Day Episode Charges and Subsequent Outpatient Visits After a Telemedicine vs In-Person Index Visit A, Charge differences were measured as telemedicine cost minus in-person cost in dollars. B, Risk ratios (RRs) were measured as the mean number of subsequent visits after a telemedicine vs in-person visit. Each condition corresponds to a unique Clinical Classifications Software Refined (CCSR) diagnostic code: obesity (END009), contraceptive and procreative management (FAC013), COVID-19 (INF012), depressive disorders (MBD002), anxiety and fear-related disorders (MBD005), neurodevelopmental disorders (MBD014), sleep-wake disorders (NVS016), other specified inflammatory conditions of skin (SKN002), respiratory signs and symptoms (SYM013), and abnormal findings without diagnosis (SYM017).

Figure 3 displays charge and subsequent-visit differences across the 10 CCSR categories. Telemedicine was associated with reduced charges across most conditions. The largest cost differences were observed for abnormal findings without diagnosis (CCSR code SYM017: −$991.55; 95% CI, −$1021.11 to −$961.99) and respiratory signs and symptoms (SYM013: −$828.78; 95% CI, −$853.23 to −$804.34). In contrast, the 3 mental and behavioral disorder categories showed comparable episode charges—depressive disorders (MBD002: −$69.47; 95% CI, −$100.90 to −$38.04), anxiety and fear-related disorders (MBD005: $38.07; 95% CI, $23.14-$52.99), and neurodevelopmental disorders (MBD014: −$28.88; 95% CI, −$54.72 to −$3.04).

The risk of follow-up visits was generally lower for telemedicine. Telemedicine for COVID-19 (CCSR code INF012) had an RR for subsequent visits of 0.35 (95% CI, 0.30-0.41). Similarly, large decreases in risk of follow-up visits were observed for contraceptive and procreative management (FAC013: RR, 0.71; 95% CI, 0.65-0.78) and neurodevelopmental disorders (MBD014: RR, 0.76; 95% CI, 0.64-0.91). Obesity (END009) showed no difference in follow-up rates (RR, 1.02; 95% CI, 0.92-1.12), and sleep-wake disorders (NVS016) showed slightly more follow-up (RR, 1.27; 95% CI, 1.18-1.37).

### Hospital-by-Condition Analysis

For each CCSR category, a stratified analysis was conducted across hospitals (eTable 7 in Supplement 1). CCH was excluded due to insufficient sample sizes for stratified comparisons. Nearly all conditions showed lower telemedicine charges, with reductions ranging from $180 to over $1100.

For INF012 (COVID-19), all hospitals showed a lower risk of subsequent visits after telemedicine (eg, RR, 0.36 [95% CI, 0.25-0.53] at PAH), suggesting telemedicine may be sufficient for managing milder respiratory conditions. Mental health diagnosis showed considerable heterogeneity. Patients at HUP consistently exhibited higher revisit risk after telemedicine for anxiety and fear-related disorders (RR, 1.39; 95% CI, 1.28-1.51) and neurodevelopmental disorders (RR, 1.67; 95% CI, 1.37-2.02), while other hospitals showed neutral or reduced risk. These patterns suggest possible site-specific differences in care pathways for mental health conditions.

## Discussion

We conducted a target trial emulation to evaluate 30-day episode charges and follow-up use differences in telemedicine and in-person outpatient visits across 10 common clinical domains at a large academic health care system. Our findings consistently showed that telemedicine compared with in-person encounters was associated with lower health care charges and reduced short-term utilization. These trends were consistent across multiple hospitals and most clinical domains, supporting the robustness of our findings.

Several factors may underlie the lower charges observed for telemedicine encounters. Differences in coding and documentation practices, visit duration, and ancillary services, such as laboratory tests or imaging orders, could contribute to the observed variation. Telemedicine encounters may also be billed at lower evaluation and management (E&M) levels or omit certain services commonly performed during in-person visits. In addition, payer reimbursement policies and patient cost-sharing obligations may vary across modalities. Our analysis excluded physician professional and facility fees, which are structurally determined by E&M coding, to ensure comparability in downstream charges and isolate differences in subsequent resource utilization rather than billing schedule artifacts. In a sensitivity analysis including both professional and facility fees in the total 30-day episode charge, results remained consistent with the primary findings.

Of note, we observed substantial heterogeneity in both financial and utilization outcomes across diagnostic domains. For example, mental health conditions such as depressive and anxiety disorders showed small or no differences in episode charges, and telemedicine visits for anxiety and fear-related disorders and neurodevelopmental disorders were associated with higher revisit rates at certain hospitals, particularly HUP, possibly reflecting site-specific clinical workflows or differences in patient triage and follow-up protocols. In contrast, conditions suited for virtual management, such as acute respiratory infections and contraceptive care, were associated with large charge reductions and significant decreases in follow-up use. Some conditions with high savings for telemedicine care, including obesity and contraceptive management, had relatively lower telemedicine uptake, highlighting opportunities to expand virtual care in areas where cost savings and efficiency gains may be substantial but where virtual care is currently underused.

Furthermore, this study demonstrates the value of applying the target trial emulation framework to clinical EHR data in operational health care evaluation. The richness of EHR data enabled comprehensive capture of visit modality (telemedicine vs in-person), structured diagnostic classifications, and linkage to detailed billing and reimbursement information, allowing for episode-level analysis of both financial and utilization outcomes. By emulating a hypothetical trial comparing telemedicine with in-person care, we minimized selection bias and allowed for robust estimation of comparative outcomes across clinically and operationally relevant subgroups. This approach is applicable for health systems seeking to monitor and optimize their care models in a dynamic clinical health care environment.

These findings have several important implications for payers, health systems, and patients. From a payer perspective, these results indicate that telemedicine does not increase overall spending on a per-episode basis and may represent a cost-efficient mode of outpatient care. From a health system perspective, however, in-person care remains the more financially advantageous setting. The overall financial impact depends on total visit volume and capacity constraints: if telemedicine increases access and total encounter frequency, aggregate system-level charges could rise despite lower per-episode charges, as suggested in prior studies.^11,14^ Health systems adopting telemedicine may therefore need to control delivery costs or redesign workflows to maintain financial sustainability while preserving the access and efficiency benefits. For patients, the reduced episode charges may reflect lower out-of-pocket spending, making telemedicine a more affordable and convenient option for managing common outpatient conditions. In addition, the availability of telemedicine itself may alter care-seeking behavior and patient preferences. By reducing indirect patient burdens such as travel time, parking fees, time off work, and scheduling constraints,^22,23^ telemedicine can prompt some patients to seek care who might otherwise have forgone an in-person visit or to become repeat telemedicine users.

Beyond financial metrics, telemedicine offers a charge-reducing and resource-efficient alternative for managing routine outpatient needs, potentially lowering operational costs and reducing unnecessary in-person visits from a care delivery perspective. The observed reduction in downstream use further indicates that when appropriately applied, telemedicine can support more targeted and effective care, countering concerns that it may lead to redundant or fragmented services. Additionally, local infrastructure and clinical workflows can influence the scalability and impact of virtual care. These insights can guide health system design and resource planning as telemedicine becomes an enduring feature of outpatient care.

### Limitations

Our study has limitations. First, the analysis was conducted within a single health system, and our future research will generalize the settings to include different patient demographics, reimbursement structures, or telehealth infrastructure. Second, while we focused on visits categorized by primary clinical concern areas, patients often present with multiple clinical issues, and the severity of these conditions may vary across visit types. Clinicians or patients may selectively choose telemedicine for less-severe conditions. Third, this analysis was based on unit costs and short-term use surrounding a single index visit. As a result, we were unable to evaluate the longer-term patterns of use. Fourth, detailed clinical or patient-reported outcomes were not available. Although 30-day subsequent visits may serve as a proxy for short-term care needs, future work linking telemedicine encounters with direct clinical outcomes will be essential to assess care quality alongside charge and utilization. Fifth, as with all observational studies, unmeasured factors may have resulted in residual confounding despite careful statistical adjustment. For example, telemedicine may be selectively used for patients with milder conditions or greater preferences for convenience or by clinicians with different follow-up or triage practices, which could contribute to the lower observed episode charges and follow-up rates even after propensity score matching. Sixth, as with all EHR-based analyses, billing and coding data may be subject to variable completeness and documentation practices, and potential differences in coding accuracy between telemedicine and in-person visits cannot be ruled out.

## Conclusions

In this comparative effectiveness research using a target trial emulation of outpatient telemedicine and in-person visits, telemedicine was associated with lower charges and reduced short-term follow-up across a range of clinical conditions. The target trial emulation framework proved useful in generating actionable insights using routinely collected health data. As health care systems continue to adapt to hybrid care models, these findings can inform operational strategies, value-based care initiatives, and reimbursement policies for integrating telemedicine into sustainable modern outpatient care.

## Notes

Supplement 1. **eTable 1.** Face-to-Face List of Encounter Types **eTable 2.** Definitions for the Top 10 CCSR Categories Included in the Analysis **eTable 3.** Study Variables **eTable 4.** Protocol Specification of Target Trial Emulation **eTable 5.** Supplemental Results: Propensity Score Weighting **eTable 6.** Supplemental Results: Including Physician and Facility Fees **eTable 7.** Supplemental Results: Subgroup Analysis by Hospitals and Conditions **eFigure 1.** Flow Diagram of Study Cohort Selection **eFigure 2.** Schematic of the Emulated Target trial **eFigure 3.** Patient Characteristic Balance Before and After Propensity Score Matching

Supplement 2.
Data Sharing Statement
