# Supplementary material for: Episode Charges and Subsequent Visits After Telemedicine vs In-Person Care
Source: JAMA Netw Open. 2026 Feb 9;9(2):e2556127. doi: 10.1001/jamanetworkopen.2025.56127 (PMC12887741; doi:10.1001/jamanetworkopen.2025.56127)
Supplement: Supplement 1. — eTable 1. Face-to-Face List of Encounter Types eTable 2. Definitions for the Top 10 CCSR Categories Included in the Analysis eTable 3. Study Variables eTable 4. Protocol Specification of Target Trial Emulation eTable 5. Supplemental Results: Propensity Score Weighting eTable 6. Supplemental Results: Including Physician and Facility Fees eTable 7. Supplemental Results: Subgroup Analysis by Hospitals and Conditions eFigure 1. Flow Diagram of Study Cohort Selection eFigure 2. Schematic of the Emulated Target trial eFigure 3. Patient Characteristic Balance Before and After Propensity Score Matching [file jamanetwopen-e2556127-s001.pdf]

Supplementary Online Content

Zhang B, Li L, Lu Y, et al. Episode charges and subsequent visits after telemedicine vs in-person care. *JAMA Netw Open*. 2026;9(2):e2556127.
doi:10.1001/jamanetworkopen.2025.56127

eTable 1. Face-to-Face List of Encounter Types

eTable 2. Definitions for the Top 10 CCSR Categories Included in the Analysis

eTable 3. Study Variables

eTable 4. Protocol Specification of Target Trial Emulation

eTable 5. Supplemental Results: Propensity Score Weighting

eTable 6. Supplemental Results: Including Physician and Facility Fees

eTable 7. Supplemental Results: Subgroup Analysis by Hospitals and Conditions

eFigure 1. Flow Diagram of Study Cohort Selection

eFigure 2. Schematic of the Emulated Target trial

eFigure 3. Patient Characteristic Balance Before and After Propensity Score Matching

This supplementary material has been provided by the authors to give readers additional information about their work.

© 2026 Zhang B et al. *JAMA Network Open*.

eTable 1. Face-to-face list of encounter types

Encounter Type	Encounter Type Name
2	Walk-In
3	Hospital Encounter
11	Research Encounter
50	Appointment
76	Telemedicine
81	Ophth Exam
91	Home Care Visit
101	Office Visit
108	Immunization
120	Endoscopy
200	Allied Health Visit
436	PT/OT/ST
438	OBGYN Visit
441	In Home Primary Care Encounter
449	PEC Visit
450	OBGYN SURGERY
452	Out of Office Visit
600	General Surgery
604	Anticoagulation Therapy
610	Infusion Visit
1003	Procedure Visit
2523	Hospice F2F Visit
32002	Care Management
32003	Virtual Visit
32006	Psych Office Visit
32009	Psych Allied Health
32012	Procedure
32013	Allied Health Visit (Non-Chargeable)
32030	Telehealth
32032	Psych Allied Health (Non-Chargeable)
304113	Palliative Home Care Visit
304114	CCBH Scheduled
304117	Telemedicine (Non-Chargeable)
1650000002	TXP Evaluation

© 2026 Zhang B et al. *JAMA Network Open*.

eTable 2. Definitions for the top 10 CCSR categories included in the analysis

The Clinical Classifications Software Refined (CCSR) for ICD-10-CM, developed by the Healthcare Cost and Utilization Project (HCUP), aggregates diagnosis codes into over 530 clinically meaningful categories across 22 body systems. These categories align broadly with chapters in the ICD-10-CM codebook and support high-level classification of healthcare utilization patterns.

In this study, we focused on the ten most common primary clinical concerns managed through telemedicine during the study period, as defined by their CCSR categories. These included:

- obesity (END009)
- contraceptive and procreative management (FACE013)
- COVID-19 (INF012)
- depressive disorders (MBD002)
- anxiety and fear-related disorders (MBD005)
- neurodevelopmental disorders (MBD014)
- sleep-wake disorders (NVS016)
- other specified inflammatory conditions of skin (SKN002)
- respiratory signs and symptoms (SYM013)
- abnormal findings without diagnosis (SYM017)

Each CCSR code falls under a broader ICD-10 chapter, briefly described below for context:

**ICD-10-CM Body System**	**Brief Description**
END (Endocrine, Nutritional, and Metabolic Diseases)	Includes 17 categories covering conditions such as thyroid disorders, diabetes mellitus, obesity, and cystic fibrosis.
FAC (Factors Influencing Health Status and Contact with Health Services)	Includes 29 categories such as aftercare, transplant status, screening, and exposure or encounters with infectious disease.
INF (Certain Infectious and Parasitic Diseases)	Captures communicable diseases including COVID-19.
MBD (Mental, Behavioral, and Neurodevelopmental Disorders)	Comprises 32 categories including disorders such as depressive disorders, alcohol-related disorders, and suicide ideation/attempt/intentional self-harm.
NVS (Diseases of the Nervous System)	Includes 22 categories such as neurocognitive disorders, Parkinson’s disease, and sleep disorders.
SKN (Diseases of the Skin and Subcutaneous Tissue)	Encompasses 7 categories including contact dermatitis and pressure ulcers.

© 2026 Zhang B et al. *JAMA Network Open*.

SYM (Symptoms, Signs, and Abnormal Clinical and Laboratory Findings, Not Elsewhere Classified)	Includes 18 categories for symptoms like fever, fatigue, nausea, or findings without a definitive diagnosis.

These ten categories served as the analytic strata for comparing telemedicine and in-person visit distributions. Additional information on the CCSR system is available from HCUP: https://hcup-us.ahrq.gov/toolssoftware/ccsr/dxccsr.jsp.

© 2026 Zhang B et al. *JAMA Network Open*.

eTable 3. Study variables

Variable	Functional form	Values	Detail
**Outcome**			
**Charges**	Numeric	-	Based on records in the amount column.
**Subsequent visits**	Integer	-	Based on records in the visit table.
**Treatment**			
**Visit type**	Indicator	Telemedicine/In-person visit	Based on the encounter type in eTable 1. The values "Telemedicine", "Virtual visit", "Telehealth", and "Telemedicine (Non-Chargeable)" is telemedicine visit, otherwise is in-person visit.
**Sociodemographic variables**			
**Age (years)**	3 categories	< 40 years<br>40-64 years<br>⩾65 years	Age is defined as the integer of (date – birth date)/365.25.
**Sex**	Indicator	Male/Female	Based on records in the sex column.
**Race/Ethnicity**	5 categories	Asian<br>Hispanic<br>NHB<br>NHW<br>Other/unknown	Based on records in the race and ethnicity columns.
**Primary insurance plan**	4 categories	Commercial<br>Medicaid<br>Medicare<br>Self-Pay/Unknown	Based on the PlanFCName column. "Commercial" includes "Managed Care", "Commercial", "Blue Cross", and "Blue Shield"; "Medicaid" includes "Medicaid", "Medicaid (MA)", and "Managed Medicaid"; "Medicare" includes "Medicare" and "Managed Medicare"; all others are categorized as "Self-Pay/Unknown". For encounters with multiple insurance records on the same visit date, a hierarchy was applied to assign a single primary category: Medicare -> Managed Medicare -> Medicaid -> Medicaid (MA) -> Managed Medicaid -> Commercial -> Managed Care -> Blue Cross -> Blue Shield -> Self-Pay/Unknown.
**Patient portal user**	Indicator	Yes/No	Based on the MyPennMedicine_Activated column.
**Marital Status**	Indicator	Yes/No	Based on the Marital_status column.
**Encounter category**	3 categories	Return patient visit<br>New patient visit<br>Unknown	Based on the enc_cat column.
**Median household income**	3 categories	<$50,000<br>$50,000 to $100,000<br>⩾$100,000	Based on patients' zip code and calculated using R package tidycensus.
**Charlson Comorbidity Index score**	3 categories	0<br>1-2<br>⩾3	Based on diagnosis domain.
**Distance from home to place of service**	3 categories	<5 miles<br>5 to 15 miles<br>⩾15 miles	Based on patients' and place of service's zip code and calculated using R package zipcodeR.

© 2026 Zhang B et al. *JAMA Network Open*.

eTable 4. Protocol specification of target trial emulation

**Protocol component**	**Hypothetical trial specification**	**Target trial emulation**
Eligibility criteria	• Outpatient setting<br>• Face-to-face visits<br>• Within ten common categories	Same
Treatment strategies	(1) Telemedicine visits<br>(2) In-person visits	Same
Treatment assignment	Randomly assigned to telemedicine or in-person visits	We assumed random assignment after propensity score matching using a list of confounders
Outcomes	(1) Episode charges<br>(2) subsequent visits	Same
Follow-up	For each visit, follow-up began from index date until 30 days after index date	Same
Causal contrasts	Intention-to-treat (ITT) effect	Same
Statistical analysis	Linear regression, Poisson regression	Same

© 2026 Zhang B et al. *JAMA Network Open*.

eTable 5. Supplemental results: propensity score weighting

**Episode charges**	**$ (95% CI)**
Telemedicine	41.18 (37.27 to 45.09)
In-person	568.11 (561.38 to 574.84)
Difference	-526.93 (-534.73 to -519.13)
**Subsequent visits**	**RR (95% CI)**
Risk ratio	0.74 (0.69 to 0.80)

© 2026 Zhang B et al. *JAMA Network Open*.

eTable 6. Supplemental results: including physician and facility fees

**Episode charges**	**$ (95% CI)**
Telemedicine	325.84 (322.59 to 329.09)
In-person	751.04 (741.97 to 760.10)
Difference	-425.20 (-434.75 to -415.64)

© 2026 Zhang B et al. *JAMA Network Open*.

eTable 7. Supplemental results: subgroup analysis by hospitals and conditions

		Difference or risk ratio (95% CI)
**END009**		
Charges	HUP	-$301.35 (-335.87 to -266.83)
	PMC	-$179.98 (-227.71 to -132.25)
	PAH	-$538.44 (-599.54 to -477.34)
	PPMC	-$499.61 (-571.00 to -428.22)
Subsequent visits	HUP	1.21 (1.06 to 1.39)
	PMC	0.81 (0.68 to 0.96)
	PAH	0.67 (0.55 to 0.81)
	PPMC	0.92 (0.69 to 1.24)
**FAC013**		
Charges	HUP	-$548.59 (-574.64 to -522.54)
	PMC	-$191.29 (-278.16 to -104.43)
	PAH	-$471.43 (-512.21 to -430.65)
	PPMC	-$1077.32 (-1190.91 to -963.74)
Subsequent visits	HUP	0.56 (0.50 to 0.63)
	PMC	1.27 (0.60 to 2.67)
	PAH	2.27 (1.93 to 2.66)
	PPMC	1.02 (0.75 to 1.38)
**INF012**		
Charges	HUP	-$514.64 (-611.73 to -417.54)
	PMC	-$268.07 (-427.81 to -108.34)
	PAH	-$381.90 (-468.10 to -295.69)
	PPMC	-$789.46 (-877.79 to -701.14)
Subsequent visits	HUP	0.32 (0.26 to 0.41)
	PMC	0.20 (0.11 to 0.37)
	PAH	0.36 (0.25 to 0.53)
	PPMC	0.48 (0.31 to 0.73)
**MBD002**		
Charges	HUP	-$31.23 (-62.55 to 0.09)
	PMC	-$137.68 (-237.29 to -38.07)
	PAH	-$483.55 (-640.86 to -326.24)
	PPMC	-$160.05 (-211.71 to -108.39)
Subsequent visits	HUP	0.85 (0.71 to 1.03)
	PMC	0.22 (0.12 to 0.41)
	PAH	0.78 (0.58 to 1.06)
	PPMC	1.00 (0.73 to 1.37)
**MBD005**		
Charges	HUP	$96.05 (80.55 to 111.55)
	PMC	-$121.22 (-171.47 to -70.98)
	PAH	-$163.19 (-200.13 to -126.25)

© 2026 Zhang B et al. *JAMA Network Open*.

	PPMC	-$221.85 (-275.24 to -168.46)
Subsequent visits	HUP	1.39 (1.28 to 1.51)
	PMC	0.36 (0.26 to 0.50)
	PAH	0.77 (0.59 to 1.01)
	PPMC	0.76 (0.59 to 0.99)
**MBD014**		
Charges	HUP	-$5.42 (-38.51 to 27.68)
	PMC	-$196.34 (-283.40 to -109.28)
	PAH	-$183.43 (-280.17 to -86.68)
	PPMC	-$173.44 (-265.32 to -81.56)
Subsequent visits	HUP	1.67 (1.37 to 2.02)
	PMC	0.08 (0.05 to 0.12)
	PAH	0.57 (0.32 to 1.04)
	PPMC	1.04 (0.62 to 1.72)
**NVS016**		
Charges	HUP	-$126.22 (-142.44 to -110.01)
	PMC	-$1101.25 (-1224.99 to -977.50)
	PAH	-$257.30 (-289.30 to -255.31)
	PPMC	-$464.92 (-538.75 to -391.09)
Subsequent visits	HUP	1.41 (1.29 to 1.55)
	PMC	0.77 (0.55 to 1.09)
	PAH	0.96 (0.77 to 1.20)
	PPMC	0.89 (0.68 to 1.17)
**SKN002**		
Charges	HUP	-$308.00 (-341.62 to -274.37)
	PMC	-$387.87 (-531.49 to -244.25)
	PAH	-$308.90 (-498.12 to -119.68)
	PPMC	-$315.44 (-369.85 to -261.02)
Subsequent visits	HUP	0.68 (0.56 to 0.82)
	PMC	0.73 (0.41 to 1.33)
	PAH	1.51 (0.58 to 3.97)
	PPMC	0.65 (0.54 to 0.79)
**SYM013**		
Charges	HUP	-$855.70 (-888.37 to -823.03)
	PMC	-$546.10 (-601.88 to -490.33)
	PAH	-$725.24 (-774.88 to -675.60)
	PPMC	-$1018.46 (-1107.38 to -929.55)
Subsequent visits	HUP	0.65 (0.58 to 0.73)
	PMC	0.79 (0.60 to 1.03)
	PAH	0.92 (0.72 to 1.18)
	PPMC	0.87 (0.69 to 1.11)
**SYM017**		
Charges	HUP	-$1149.79 (-1193.80 to -1105.79)
	PMC	-$524.25 (-580.24 to -468.25)
	PAH	-$793.49 (-859.32 to -727.67)

© 2026 Zhang B et al. *JAMA Network Open*.

	PPMC	-$850.87 (-909.06 to -792.68)
Subsequent visits	HUP	0.84 (0.74 to 0.97)
	PMC	0.87 (0.63 to 1.22)
	PAH	0.88 (0.69 to 1.10)
	PPMC	0.91 (0.74 to 1.11)

© 2026 Zhang B et al. *JAMA Network Open*.

eFigure 1. Flow diagram of study cohort selection

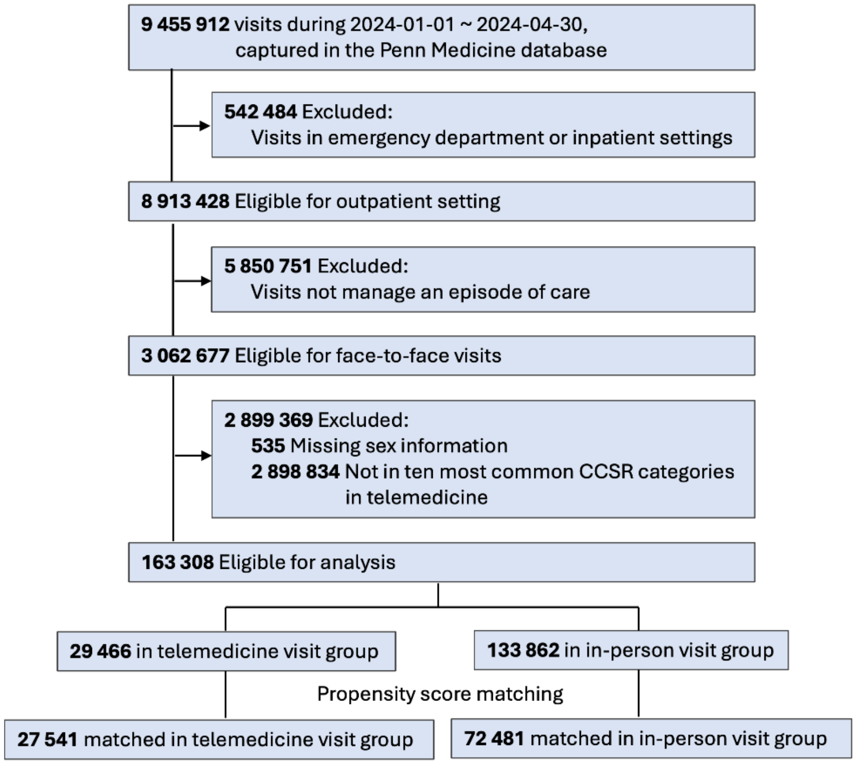

© 2026 Zhang B et al. *JAMA Network Open*.

eFigure 2. Schematic of the emulated target trial

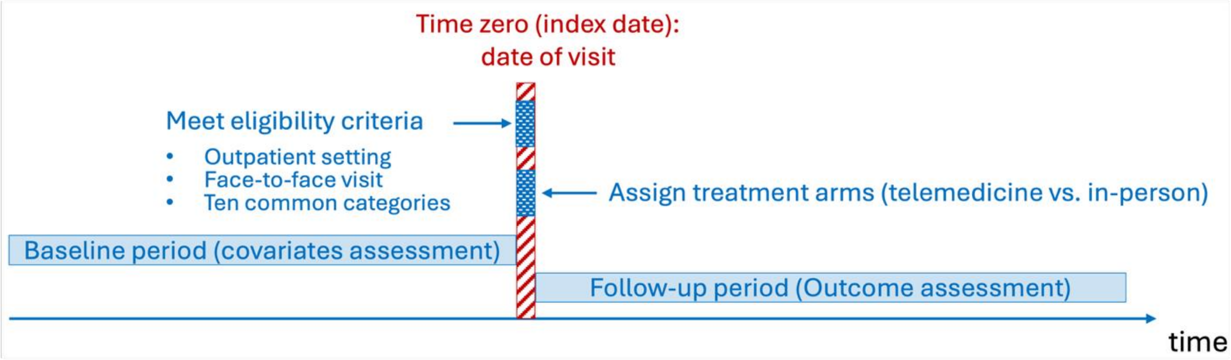

© 2026 Zhang B et al. *JAMA Network Open*.

eFigure 3. Patient characteristic balance before and after propensity score matching

The upper panel displays the top 20 covariates with the largest standardized mean differences (SMDs) before matching, and the lower panel displays the top 20 covariates with the largest SMDs after matching.

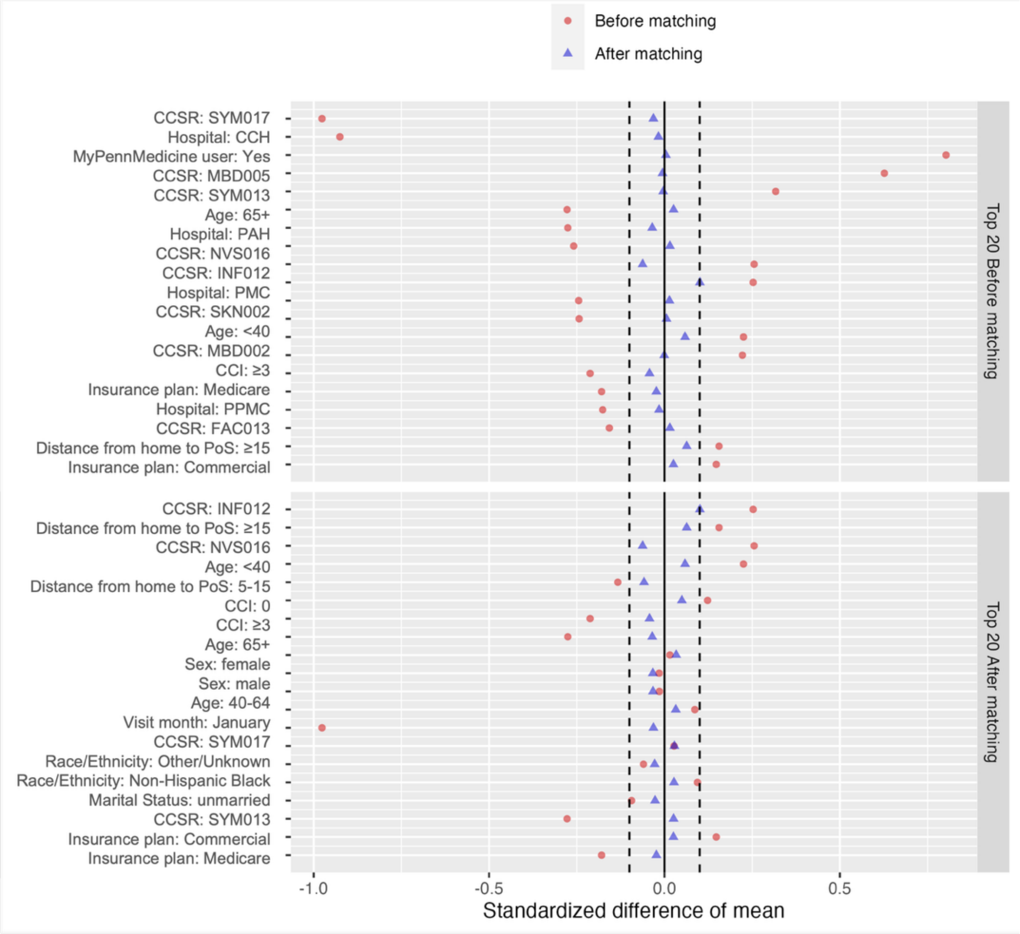

© 2026 Zhang B et al. *JAMA Network Open*.
