# Supplementary material for: Episode Charges and Subsequent Visits After Telemedicine vs In-Person Care
Source: JAMA Netw Open. 2026 Feb 9;9(2):e2556127. doi: 10.1001/jamanetworkopen.2025.56127 (PMC12887741; doi:10.1001/jamanetworkopen.2025.56127)
Supplement: Supplement 2. — Data Sharing Statement [file jamanetwopen-e2556127-s002.pdf]

Data Sharing Statement

Zhang. Episode Charges and Subsequent Visits After Telemedicine vs In-Person Care. *JAMA Netw Open*. Published February 09, 2026. doi:10.1001/jamanetworkopen.2025.56127

Data
Data available: No

Additional Information
Explanation for why data not available: The results reported in this study are based on detailed individual-level patient data. The datasets generated and analyzed are not publicly available due to the University of Pennsylvania's privacy and confidentiality requirements.
